# How Do Mobile Social Apps Matter for College Students’ Satisfaction in Group-Based Learning? The Mediation of Collaborative Learning

**DOI:** 10.3389/fpsyg.2022.795660

**Published:** 2022-02-25

**Authors:** Xuyan Wang, Renyu Zhang, Xiaojiong Wang, Dongming Xu, Fangqing Tian

**Affiliations:** ^1^The College of Management and Economics, Tianjin University, Tianjin, China; ^2^PowerChina Leasing Co. Ltd., Beijing, China; ^3^Institutes of Science and Development, Chinese Academy of Sciences, Beijing, China; ^4^Business School, The University of Queensland, Brisbane, QLD, Australia

**Keywords:** mobile social apps, knowledge sharing, collaborative learning, course satisfaction, structural equation model

## Abstract

Recently, many universities apply mobile tools to teaching practices. For instance, some teachers may set up groups on mobile social apps and assign course tasks and advise college students to submit papers online. Nevertheless, how these mobile social apps affect teaching practices, especially the process of students’ satisfaction needs to be further explored. To fill this research gap, we build a theoretical model of how mobile social apps’ functions affect course satisfaction from the perspective of Media Richness theory and the Uses and Gratifications (U and G) theory. A total of 186 valid questionnaires from college students in China were collected, and a structural equation model was built to test our research model. The results show that as: (1) only the communication function has positive impacts on knowledge sharing, while the impact of the information storing function and information distribution function on knowledge sharing is not significant; (2) knowledge sharing does not affect course satisfaction in a direct way, but it can act indirectly through promoting collaborative learning, which shows the mediating role of collaborative learning. The theoretical implications and practical implications of the study are discussed.

## Introduction

With the rapid growth of the Internet and information technology, mobile devices, such as smartphones, are becoming more and more popular. Contemporary young people are keen to use mobile social apps, which have become a common phenomenon in their work and life (De Zuniga, 2012; Chen et al., 2015; Greenwood and Gopal, 2015; Miranda et al., 2016). Given the rapid development of mobile devices, mobile social apps have become more convenient than previous social media, and their functions are more advanced and smart (Schlagwein and Hu, 2017; Moravec et al., 2019). More importantly, the sudden appearance and spread of COVID-19 in 2020 not only affected people’s work and life, but also affected students’ educational practice activities and accelerated the digital transformation of the education industry. However, this large-scale online education practice shows that the development of digital education also needs a good platform as a basic support (Lyons et al., 2021; Wang et al., 2021). For example, the mobile social apps, such as Tencent meeting, QQ group, WeChat group, and DingTalk (the above are popular mobile social apps in China) used by students, have promoted the use of class learning exchange for a group. As a result, telework is becoming more and more feasible, making commuting to an office or factory unnecessary. What is more, the emergence of COVID-19 has accelerated the transformation of this type of work and study mode (Malhotra, 2021). Thus, how to promote the continuous and in-depth development of digital education in the “post-epidemic era” is an issue that people should continue to pay attention to. The online and offline hybrid approach makes it urgent to redesign courses using digital technologies, such as social media. To take an example, it is necessary to emphasize online and offline real-time interaction and collaborative learning for improving learning efficiency (Stahl, 2021).

With regard to the role of social media in education, a number of scholars have studied how students should choose media that can be more conducive to mobile learning for different levels of media richness (Herrero et al., 2015; Tseng et al., 2016). Some scholars have pointed out that easy-to-use apps are one of the technical factors that affect student satisfaction (Bekele, 2010). Taking the perspective of integrating elaboration likelihood model and social media capabilities, Zhang et al. (2019) explored how social apps help reshape students’ attitudes about business ethics. The characteristics of the mobile social apps, such as ubiquity, access, richness, and flexibility, give students the ability to connect with active instruction anytime, anywhere. Students can also post, comment on, and share information regardless of geographic location or time. In the end, this enlarges access to education for all, promotes collaboration among students, and extends learning beyond the classroom. Students’ collaborative learning includes conversation and data sharing; students can obtain their important resources by interacting with others more frequently (Tseng et al., 2016). Mobile social apps can enable students to benefit from social interactivity and the connective learning process, which will also foster their performance and satisfaction in terms of learning content. Mobile learning is a focus of flexible use of various learning sources in learning environments (Aparicio et al., 2016). However, how mobile social apps affect the educational effectiveness needs to be further explored. Therefore, there are two research gaps. First, in the context of group learning and teaching, the influence of the use of mobile social apps on the learning outcome (that is, satisfaction) perceived by students is unclear. Second, given that social media has multiple functions, how does the specific function of mobile social apps affect this process?

This paper intends to answer the above questions from the perspective of media richness and Uses and Gratifications (U and G). From the perspective of U and G, mobile social apps can provide instant feedback and multiple clues between different individuals; they have the potential to meet people’s basic needs for connecting with others. For example, if students need to connect with others instantly, they will choose a social tool, such as a common mobile social app, and use it actively to meet their needs (Chen, 2011). In addition, the search for personal satisfaction may be related to media richness. Media richness theory posits that the audience’s understanding is related to the different degrees of media richness (Alamäki et al., 2019). The richer the media, the greater the ability of the media to convey information. In other words, rich media have greater potential in meeting individual needs, such as information needs and social needs, which can easily make people feel satisfied. Therefore, combining media richness theory with user motivation derived from the perspective of U and G can enhance students’ understanding of the reasons for this media use and subsequent changes in attitudes and behaviors (Ishii et al., 2017).

**Figure 1:**
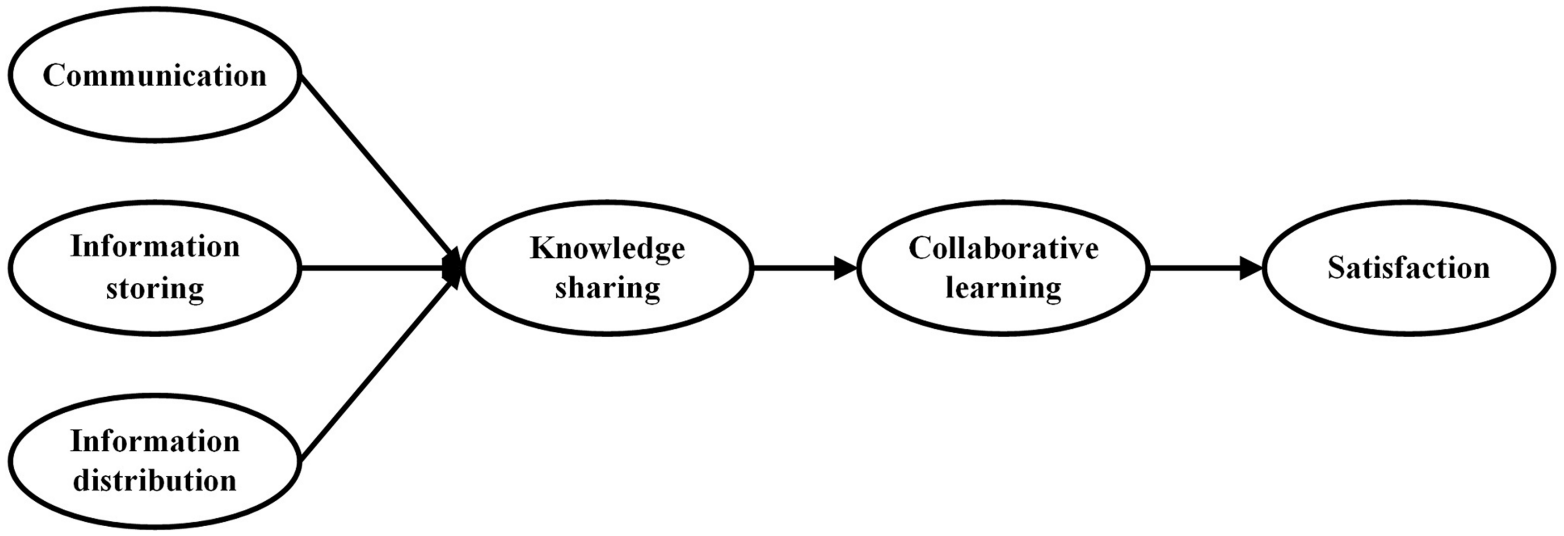
Theoretical model.

The online environment provides students with the opportunity to actively participate in the learning process and meet their learning needs. The technical support of the corresponding tools can help students transfer information from many aspects, enhance students’ understanding of information, and make knowledge explicit and share it. That is, the functions of the mobile social apps can provide more convenient task information exchange and meet students’ task information needs, thereby helping them complete their learning tasks. According to media richness theory, by matching the media with students’ task information needs, the efficiency of communication between students and other participants can be improved, and the choice of different media is likely to create different learning experiences for students. Therefore, drawing from the media richness and U and G theory, we propose a theoretical model (shown in Figure 1) to explore what and how system characteristics of mobile social apps affect students’ knowledge sharing behavior and to investigate the effect of knowledge sharing caused by mobile social apps usage on course satisfaction through collaborative learning.

## Theoretical Background and Research Model

### Media Richness Theory

Media richness theory refers to the ability of different degrees of media to convey information and knowledge (Daft and Lengel, 1986; Cooper and Cooper, 2003; Balaji and Chakrabarti, 2010; Alamäki et al., 2019). Initially, when managers of enterprises need to choose the right media, this theory was used to explore how the richness of information of different media would affect the choice. Then, scholars extended the theory to multiple fields, such as tourism, human resource management, and economics (Allen et al., 2004; Alamäki et al., 2019). Media richness theory claims that the higher the richness of the media, the stronger its ability to provide information and clues (Daft and Lengel, 1986; Shiue et al., 2010; Klitmøller and Lauring, 2013). Communication between individuals often uses media to convey information, such as face-to-face communication and social media. Richer media contain more diverse modes of communication; for example, mobile social apps can transmit information through text, pictures, audio, video, etc. Therefore, the richer the media is, the higher the ability to convey information, which can help individuals understand information and communication better (Koo et al., 2011).

With the rapid development of information technology, mobile learning has also become a new trend in the field of education, and a better understanding of how to effectively use social media is important for improving the efficiency of mobile learning. For instance, Ogara et al. (2014) investigated the influencing factors of mobile instant messaging with medium media richness, in terms of social presence and user satisfaction. Based on 239 questionnaires from college students, the results showed that perceived richness is one of the critical drivers of social presence in mobile instant messaging, which in turn promotes user satisfaction. Furthermore, Lu et al. (2014) studied the impact of websites on college students’ sports activities from the perspective of high media richness and high interactivity features. It was found that media richness could change students’ intention to visit fitness centers, through the mediating effects of knowledge, attitudes, and integrity. Their research would be beneficial for schools to design interventions in network health based on this feature. Research has pointed out that using relatively new social media to communicate is more effective than using traditional social media. The newer version of social media is more comprehensive in information expression, and it contains functions that can help students correctly deal with the uncertainties faced in learning tasks (Lai and Chang, 2011). Other scholars have evaluated the media richness of various message delivery methods in the mobile learning environment based on the media richness theory, and adopted appropriate information delivery media to support the corresponding learning activities in the mobile learning environment, aiming to provide a system for the mobile learning environment (Lan and Sie, 2010). Park et al. (2012) explored three media richness text-based social media from a media richness theory perspective: email, text message, and Facebook wall. When matching among technical characteristics, technology-based goals and social environment, users could choose the given technology better. It can be seen that media attributes remain an important aspect of individual attitudes and behaviors. The theory of media richness proposes that the immediacy of information conveyed through the media can enable students to receive information smoothly and understand the central idea more specifically (Zhang et al., 2019). As it can be seen, scholars have begun to focus on those technology-advanced media like mobile social apps, trying to expand the boundaries of media richness theory.

### The U and G Theory

The U and G theory explains the audience’s motivation to use media and what needs to be satisfied, from the perspective of the audience (Mondi et al., 2008; Ersoy and Guneyli, 2016). It is believed that the audience can control the process of media communication through active use of media, and the use of media was mainly for individual needs and willingness (Mondi et al., 2008; Raacke and Bonds-Raacke, 2008). In other words, the audience may choose to use a certain media for psychological reasons and motivations (Ko et al., 2005; Choi et al., 2016). When their own needs are met, the audience will perceive gratifications through the use of media.

This theory made people realize that different audiences have different purposes when using the same media. How individuals use media are the focus of U and Gs theory, so it highlights the importance of individuals in the process. The media should be able to meet the needs of these various audiences, for example, cognitive needs and social needs (Mondi et al., 2008; Ersoy and Guneyli, 2016). Research by Mueller et al. (2011) validated that different types of satisfaction affect users’ use of some network-based media. When functional needs, emotive needs, and contextual needs are satisfied, users would be more involved in virtual knowledge communities. Some scholars believe that, as a theoretical framework, the U and G theory focuses on explaining how people use media to meet their social and psychological needs, and to determine the consequences of these needs. U and G posits that different tools with diversified functions will attract the attention of users, and the audience will choose the media that meets their needs best. According to 18 interviews, Gan and Wang (2015) revealed what satisfaction users will get from the use of Weibo and WeChat, including: content gratification, social gratification, and hedonic gratification. Users could get content satisfaction due to obtaining high-quality information and maintain individual social networks conveniently to obtain social satisfaction. In addition, users will also use such social media for entertainment and to meet the need for hedonic. It can be seen that how the individual uses the media and the degree of satisfaction of this media to the individual’s needs in the course of use will affect the formation of individual attitudes (Bae, 2018). In the context of the education, there are inconsistent findings about which needs of students can be met by high rich media. Take a situation in which instant messaging may more satisfy the social needs of college students, sometimes it may be unclear in learning needs. Vrocharidou and Efthymiou (2012) found in online entrepreneurship courses that mobile social media (Line, Facebook, and WeChat) can meet their social needs as well as their academic learning needs. The main reason is that such digital technology can promote the interaction between learners and the course content, increase learners’ enthusiasm, and increase their participation in entrepreneurial knowledge acquisition. Therefore, it is necessary to verify how the functions of mobile social apps on earth meet student’s needs in courses.

### Mobile Social Apps and Knowledge Sharing

The emergence of mobile social apps opened up channels to connect people, which breaks the limitations of traditional media in terms of time and geography. The pervasive usage of various social apps (like Facebook and WeChat) improves the flow and timeliness of information greatly (Liu et al., 2009; Tseng et al., 2016). Some features of mobile social apps make users willing to use them, such as searching for information, communicating, and getting entertainment. According to Zhang et al. (2012), the functions of mobile social apps include communication, information storing, and information distribution. Users can communicate anytime and anywhere when using mobile social apps, which is very convenient. At the same time, the information storing and information distribution functions of mobile social apps enable users to view and retain past dialogue information so that it can be reviewed later. By storing the existing information in a classified and orderly way, users can extract the information they need easily. There is no doubt that the time taken to extract information will be reduced, and the efficiency of information exchange and delivery will be improved. Nowadays, mobile social apps introduce this emerging technology, which can intelligently classify and store the information that has been collected and processed, which can ensure that users can extract it at any time when needed, and improves the efficiency and utilization of information transmission. It is worth noting that some studies have shown that, considering the diversity of users and the heterogeneity of different types of social media, it is necessary to distinguish the characteristics of social media and give them exclusive attention (Kim, 2014). That is, it is of great significance to distinguish the specific functions of mobile social apps and to explore its specific impact on individual attitudes and behaviors from the perspective of this theory.

Knowledge sharing refers to the process of exchanging knowledge between individuals and groups, one of the main contents of knowledge management (Davenport and Prusak, 1998; Chang and Chuang, 2011). In the organizational context, knowledge sharing is not only an important means and driving force for knowledge innovation, but also a catalyst for promoting the value of knowledge (Xiao et al., 2017). Many scholars have explored factors that motivate employees to share knowledge, including individual psychological characteristics, organizational culture and organizational structure, social capital and human resource management, technical characteristics, and so on (Wang and Noe, 2010; Rudall, 2012; Yen, 2015; Zhao et al., 2020). By sharing information or know-how they have acquired, individuals help other members or work with others to solve problems together. Aware of the significance of knowledge sharing, some scholars extended the concept of knowledge sharing to employees, teams, department, and even the entire organization, exploring ways and means of transferring or re-disseminating knowledge to other parts through various methods and channels (Huysman and Wulf, 2006). This is designed to enable knowledge recipients to maximize access to and use of knowledge so that they can better accomplish their tasks (Qureshi et al., 2018). Although the research on knowledge sharing has now expanded to the online scope, it is still a means for a certain group to achieve the ultimate goal (Jiménez-Zarco et al., 2015).

Mobile social apps are powerful tools that facilitate the development of the important processes of knowledge sharing by promoting communication and interaction within student groups and other users (Jiménez-Zarco et al., 2015). When using mobile social apps, more students can be involved in communication and interaction because of the convenience of communication (Zhang et al., 2012; Wang et al., 2020; Chen and Tsao, 2021). Student users who use mobile social apps in the education field also have cognitive needs, such as expressive information sharing (Yen, 2015). Due to the information distribution function of mobile social apps, students can be more involved in the process of information distribution. It can help them obtain and correct new information in time, improving the timeliness and accuracy of the information. The information storing function enables students to classify and store the existing information in an orderly manner, and to extract the required information easily. It reduces the cost and time of information extraction greatly, and speeds up the efficiency of information exchange and transfer. Students can share their different ideas and other key information; such as course data by these apps when they want to learn something new. Therefore, these three functions of mobile social apps will have positive impacts on knowledge sharing. The following hypotheses are proposed as:

***Hypothesis 1a***: The communication function of mobile social apps has a positive impact on knowledge sharing.

***Hypothesis 1b***: The information distribution function of mobile social apps has a positive impact on knowledge sharing.

***Hypothesis 1c***: The information storing function of mobile social apps has a positive impact on knowledge sharing.

### Knowledge Sharing, Collaborative Learning, and Satisfaction

The concept of knowledge sharing has such great research value because it can bring about positive effects (Lin et al., 2010; Rasheed et al., 2020). Majchrzak et al. (2004) pointed out in their study of American companies that knowledge sharing can positively influence the innovation activities of enterprises. It was found that active and effective knowledge sharing can promote the performance and innovation of members of an organization. In a survey of 125 employees in tele-communications companies, Aulawi et al. (2009) found that active and effective knowledge sharing within the enterprise can promote individuals to generate a wider range of creative ideas and opportunities to develop new businesses, thereby enhancing innovation capabilities. When students use mobile social apps to interact and share information, the higher the degree of knowledge sharing, the more professional knowledge relevant to their course they learn, thus promoting their satisfaction with the course (Lee et al., 2011; Herrero et al., 2015; Tseng et al., 2016).

In college students’ courses, some tasks often require joint efforts among members; that is, they involve cooperation and collaboration. Collaborative learning occurs when students discover, understand, and build knowledge through “mutual cooperation” to achieve common goals (Kuo et al., 2017; Chis et al., 2018; Lyons et al., 2021). The process of collaborative learning is usually in a small group; each member is responsible for a part of the work and ultimately for a large learning task (So and Brush, 2008). Team members can share task-related information with each other, which can increase the diversity of knowledge. Each member can receive more information and knowledge through this process and the sharing behavior. In addition, online education has become normalized due to the epidemic and the progress of mobile technologies, which makes the role of collaborative learning more significant (Stahl, 2021).

Mobile social apps can expand students’ sources of knowledge and support collaborative learning by enhancing knowledge acquisition, knowledge sharing, and reflection processes (Liaw et al., 2008; Kuo et al., 2017). Therefore, when the level of knowledge sharing among members is higher, the more information and resources will be transmitted among members, and the interaction between them will be strengthened; this will help to improve the effect of collaborative learning (So and Brush, 2008; Coetzee et al., 2018; Roldan-Alvarez et al., 2020). Thus, collaborative learning can be helpful to promote task goals of a group or team. According to Collazos et al. (2002), one of the evaluation factors in the collaborative learning process includes performance. It can be seen that collaborative learning may bring positive learning results, such as learning course satisfaction. In addition, some researches also points out the importance of sharing and understanding among multiple individuals in collaborative learning. Therefore, it is speculated that collaborative learning may play an mediating role in the relationship between knowledge sharing and students’ course satisfaction (Stahl, 2005; Agredo-Delgado et al., 2021). Through collaborative learning, students’ thinking ability, communication ability, and teamwork skills will be improved by the end of the course, which will have a positive effect on their long-term development. That is, students will have higher satisfaction with the course (McFarland and Hamilton, 2005; Paechter et al., 2010). The following hypothesis is proposed as:

***Hypothesis 2***: Collaborative learning mediates the relationship between knowledge sharing and course satisfaction.

## Materials and Methods

### Participants and Procedure

To verify this framework, an online questionnaire was conducted. We collected data in a course taught for first-year postgraduates from a top university in China. It was suitable for two reasons. First, it is strongly encouraged to use mobile social apps (like DingTalk) in this course. The teacher will set up a group for all students who choose the course, and post important notices and task requirements online. When students have questions at any time, such as not understanding the task requirements, the teacher or teaching assistant will reply to the information in the group immediately. Second, this course focuses on group-based training. Students are required to use at least one big data technology, to analyze and solve business problems in the form of cases. However, the students who choose this course come from different professional backgrounds (finance, business administration, management science and engineering, etc.), and the task load of this course is relatively large, so it is difficult to complete a case analysis report alone. Therefore, the course tasks are required to be completed collaboratively in the form of group, that is, each group jointly completes a case analysis report. The course teacher provides the students with a range of research topics, and the students can propose specific research directions taken into account their own interests and feasibility. For example, analyzing the security of listings in Airbnb (a vacation rental platform) using a machine-learning method and establishing visual security indicators. In addition to checking the research directions proposed by the students, the teacher will also provide professional guidance on the whole process of completing the course tasks.

Collaborative learning should be a group of people working to achieve a common goal, and effective communication is an important basis for realize cooperation (Agredo-Delgado et al., 2021). In order to achieve the best group learning effect (Collazos et al., 2002), first of all, the teacher established a group for all students who choose the course through a mobile social app (such as DingTalk) at the beginning of the course to facilitate timely release of important notices and task requirements. Whenever students have questions about course-related issues, for example, when they cannot fully understand the task requirements, the teacher and teaching assistants will respond immediately. Therefore, all the students were first randomly divided into several groups, each with about 10 people. Each group needed to select a team leader and break down the tasks in more detail so that each member can be assigned to one or two small tasks. That is, allocating tasks reasonably. For example, two members were responsible for investigating possible case study topics, three members were responsible for using big data analysis technology to obtain and analyze case-related data, two members were responsible for writing case reports, two members were responsible for PowerPoint PPT production of case reports, and the last member was responsible for oral presentations of case reports. In this way, each team can cooperate to complete the task of the whole case analysis report. Because some students who chose this course might have no technical foundation at all, the teaching assistant artificially adjusted the results of the first grouping. So that each group can have members with good data analysis foundation. Each group established an online group through a mobile social app to realize task assignment and further communication. In the submitted case report, the work content of each member needs to be described by the team leader, that is, how the cooperation between members is realized and completed. The teacher will evaluate the work performance according to the completion quality of the case report and the role of each student. Therefore, this is a suitable solution for our research.

**Figure 2:**
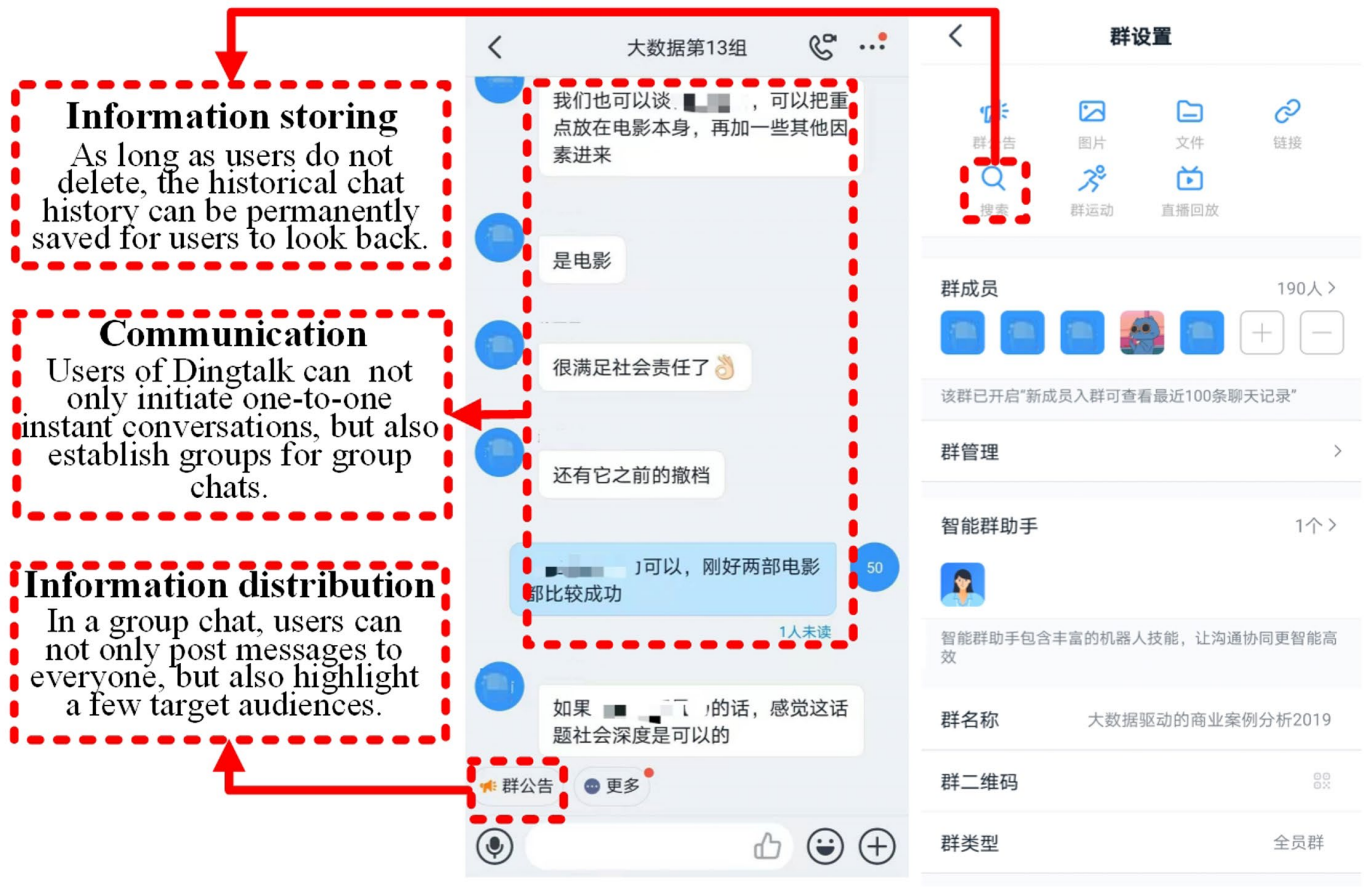
An example of group chats in a mobile social app.

The mobile social app used in this course is DingTalk, a business-oriented social app for organizational communication and collaboration. This app provides very useful functions to help users communicate and work in two ways: (1) Users of DingTalk can not only initiate one-to-one instant conversations, but also establish groups for group chats. Meanwhile, message senders can know whether the recipients have read information. (2) In a group chat, users can not only post messages to everyone, but also highlight a few target audiences. As long as users do not delete it, the historical chat history can be permanently saved for them to look back at. Figure 2 provides an example of group chats on a mobile social app page. After ensuring that students make full use of the app, we sent an online questionnaire to all of the students in the DingTalk group in the last class (197 students), and 186 valid questionnaires were collected after deleting incomplete data (94.4%).

### Variables and Measures

The measurements of all variables in this study are derived from existing research and items have been modified and deleted based on the research background. Detailed items can be found in the Appendix A. The measurement items in the questionnaire were all measured using the Likert scale (1—strongly disagree; 7—strongly agree).

#### Mobile Social Apps Functions

As per the above, our research describes the functions of mobile social apps, based on communication, information storing, and information distribution. Using mobile social apps to communicate with classmates will support students to speak freely and engage in dialogues. In this process, more diversified and rapid information is exchanged and communicated. However, traditional media usually take days, weeks, or even months to produce. In contrast, mobile social apps have the function of information distribution at any time. They also enable message senders to update changes anytime and anywhere. And, when using mobile social apps, students can store their own notes, PowerPoint PPT photos taken in class, or recordings of lectures on their mobile phones in a variety of forms when needed, or share them with others. The apps also allow them to review and consolidate the information they call up afterward and avoid forgetting it. The scale of communication, information storing, and information distribution is adapted from Zhang et al. (2012), which is used to measure the use and perception of students regarding the system characteristics of mobile social apps.

#### Knowledge Sharing

In our study, knowledge sharing refers to the process of college students sharing knowledge with other students through mobile social apps. Regarding the knowledge imparted by the course teacher, students can share their own notes and so on with other classmates; in group work, students can also share their extracurricular learning knowledge. In addition, it is also a kind of knowledge sharing when students talk about and discuss things on mobile social apps. The scale of knowledge sharing was adapted from Xiao et al. (2017) to measure the degree of knowledge sharing among students.

#### Collaborative Learning

Collaborative learning refers to students’ working with their learning partners or forming study groups to accomplish the course tasks together. Many students may not be able to fully absorb and understand the knowledge taught in the course, or some students might not understand accurately, so they find a suitable person to be their learning partner, or several students come together to form a study group. After each class, the group can discuss and study the content, and they can also impart their own course-related self-knowledge to the rest of the group. Through such a learning form, the efficiency of overall task completion can be significantly improved. The scale of collaborative learning is adapted from So and Brush (2008) to measure the occurrence between college students and their course satisfaction.

#### Course Satisfaction

Course satisfaction refers to a state of mind, to the students’ subjective evaluation of the learning experience of the whole course. When students choose a course, they usually expect to get some kind of benefit from it, such as learning knowledge and socializing. When their overall expectations of the course are satisfied, this indicates that the course satisfaction level is high. The items of course satisfaction are adapted from So and Brush (2008) to measure the occurrence between college students and their course satisfaction.

## Analysis Results

A structural equation model (SEM) is chosen in this study; there are several reasons for its advantages (Saris et al., 2009; Youssef et al., 2017). First, it can handle multiple dependent variables simultaneously. Second, it allows the existence of measurement errors. Third, factor structure and factor relationships can be estimated simultaneously. Fourth, it allows for greater flexibility of the measurement model. Finally, the structural equation can estimate the degree of fit of the entire model. SPSS 22.0 and AMOS 24 software were used to analyze and process the data.

### Measurement Model Test

In the factor analysis approach in SEM, first we used confirmatory factor analysis to examine construct validity. The scale of all of variables was included in the confirmatory factor analysis. When evaluating the reliability of the scale, we used the Cronbach’s Alpha as a standard. The results showed that the Cronbach’s Alphas of all variables are all greater than .89, which means its internal consistency is suitable. Then, when evaluating the validity of the scales, we used the composite reliability (CR) and average variance extracted (AVE) to measure convergent validity. The higher the CR value, the higher the internal consistency of each measurement model. Hair et al. (1995) believe that, in order to meet the internal consistency requirements of the measurement model, the CR threshold should be set above 0.7, and the ideal value of AVE should be above 0.5. The results in Table 1 show that the CRs are all above 0.92, all of which is much greater than 0.7, and the AVE is between 0.702 and 0.965, which are both greater than 0.5. These indicators indicate that the aggregation efficiency of the scale is good. The results in Table 2 indicate that the scale has adequate discriminant validity. Among all six variables, there are 25 measurement indicators, and the factor load of these 24 measurement constructs under the corresponding variables is above 0.7; meanwhile, the AVE of 0.5 is also greater than its correlation coefficient with other variables.

**Table 1:** Reliability and validity test.

Variable	Item	Loading	Cronbach’s alpha	Composite reliability	AVE
Communication	CM1 CM2 CM3	0.943538 0.941106 0.945764	0.93833	0.960485	0.890137
Information storing	IS1 IS2 IS3	0.978656 0.978488 0.981615	0.978989	0.986158	0.959591
Information distribution	ID1 ID2 ID3	0.98349 0.983483 0.980023	0.981867	0.988047	0.964978
Knowledge sharing	KS1 KS2 KS3 KS4 KS5	0.810787 0.907171 0.877649 0.857187 0.902021	0.920527	0.940443	0.759803
Collaborative learning	CL1 CL2 CL3 CL4 CL5	0.891332 0.87305 0.918901 0.907411 0.857758	0.934174	0.950079	0.792042
Satisfaction	SF1 SF2 SF3 SF4 SF5	0.801247 0.892428 0.889741 0.799249 0.802138	0.893567	0.9217	0.702457

**Table 2:** Discriminant validity.

	Communication	Information storing	Information distribution	Collaborative learning	Satisfaction	Knowledge sharing
Communication	**0.943471**					
Information storing	0.446013	**0.943471**				
Information distribution	0.426506	0.657472	**0.979587**			
Collaborative learning	0.541906	0.326159	0.328856	**0.982333**		
Satisfaction	0.548338	0.369914	0.338886	0.841403	**0.838127**	
Knowledge sharing	0.509297	0.309645	0.263805	0.862115	0.760883	**0.871667**

The bold values indicate the square root of AVE.

### Hypothesis Model Test

**Figure 3:**
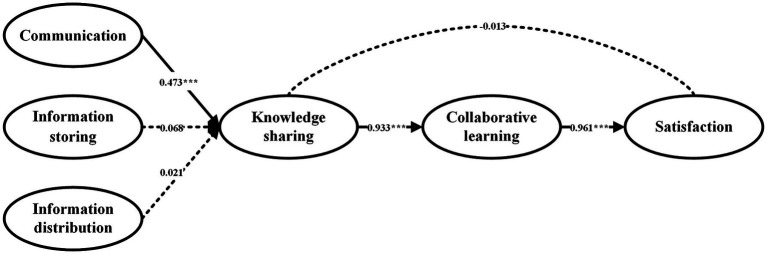
Data analysis results. ^***^p < 0.001.

The hypothesis test results of the mode in the model are shown in Figure 3. As can be seen, among the three functions of mobile social apps (communication, information storing, and information distribution function), only the relationship between communication function and knowledge sharing was significant (*β* = 0.473, *t* = 5.401, *p* < 0.001). The impact of information storing and information distribution on knowledge sharing was not significant at all (*β* = 0.068, *t* = 0.779, *p* > 0.1; *β* = 0.021, *t* = 0.247, *p* > 0.1). Therefore, hypothesis 1a was supported; hypothesis 1b and hypothesis 1c were not supported.

When validating the relationship between knowledge sharing and course satisfaction, the estimated result of these two variables (*β* = −0.013, *t* = −0.076, *p* > 0.1) was non-significant, which showed that hypothesis 2 was not supported. In other words, knowledge sharing could not directly affect satisfaction. In contrast, there was a significant positive relationship between knowledge sharing and collaborative learning (*β* = 0.933, *t* = 16.868, *p* < 0.001), and a significantly positive relationship between collaborative learning and course satisfaction (*β* = 0.961, *t* = 5.243, *p* < 0.001). That is to say, course satisfaction could be affected by knowledge sharing through collaborative learning.

### The Mediation Effect of Collaborative Learning

In order to study the mediating effect of collaborative learning between knowledge sharing and course satisfaction, additional regress analysis was performed. The analysis results are shown in Tables 3 and 4.

**Table 3:** Direct relationship between knowledge sharing and satisfaction.

The direct effect of knowledge sharing on satisfaction					
Effect	*SE*	*t*	*p*	LLCI	ULCI
Knowledge sharing→Satisfaction	0.0812	1.7079	0.0894	−0.0215	0.2989

**Table 4:** Mediating effect of collaborative learning.

The indirect effect of knowledge sharing on satisfaction				
Effect	Boot	*SE*	BootLLCI	BootULCI
Knowledge sharing→Collaborative learning→Satisfaction	0.6445	0.1138	0.4598	0.9141

It can be seen from the data in Table 4 that the interval of LLCI and ULCI (−0.0215–0.2989) contained 0, indicating that there was no significant direct relationship between knowledge sharing and course satisfaction, and then, Table 5 showed that the interval of LLCI and ULCI (0.4598–0.9141) did not contain 0, indicating that there was a significant indirect relationship between knowledge sharing and course satisfaction. This shows that collaborative learning has a complete mediating effect between knowledge sharing and course satisfaction. Therefore, hypothesis 2 was supported.

**Table 5:** Overall results.

Hypothesis		Result
H1a	Communication function of mobile social apps have a positive impact on knowledge sharing.	Supported
H1b	Information storing function of mobile social apps have a positive impact on knowledge sharing.	Not Supported
H1c	Information distribution function of mobile social apps have a positive impact on knowledge sharing.	Not Supported
H2	Collaborative learning mediates the relationship between knowledge sharing and course satisfaction	Supported

## Discussion

### Findings

Through the SEM built in this study and data analysis, the proposed hypotheses have been verified. First, the results show that only the communication function of mobile social apps had a significant positive effect on knowledge sharing of college students. However, the relationship between another two functions—information storing and information distribution with knowledge sharing—was not significant. In detail, the communication function of the mobile social apps used by the students can effectively promote the knowledge sharing process between them, which is consistent with the research of Zhang et al. (2012). The other two functions of mobile social apps are not as effective as the communication function in promoting knowledge sharing. This may be because, although all of these three functions in the mobile social apps can convey information in real time, the communication function is more effective in terms of information exchange. Due to the communications in the mobile social apps are mainly two-way transmission of information, the degree of information exchange is high. That is, continuous information exchange will promote knowledge creation and knowledge sharing. Therefore, compared with the communication function, the effects of information storing and information distribution function are weak in promoting knowledge sharing. Second, knowledge sharing does not affect course satisfaction in a direct way, but it can act indirectly through promoting collaborative learning. In other words, collaborative learning plays a key transfer role between knowledge sharing and course satisfaction, which is consistent with previous studies (Paechter et al., 2010; Kuo et al., 2017; Coetzee et al., 2018). The overall results of the hypotheses are shown in Table 5.

### Theoretical Contributions

This study explores the influence mechanism of mobile social apps functions on students’ course satisfaction from the perspective of media richness theory and U and G theory. There are three theoretical implications.

Firstly, this study integrates media richness theory and U and G theory to explain the process of how students achieve course satisfaction through mobile social apps. Previous studies have rarely explored the practical effects of mobile social apps in group learning, especially from the perspective of user needs. Because of the psychological motivations and demands at the individual level, students use mobile social apps with high media richness to communicate and study (Del Barrio-Garcia et al., 2015). Our findings indicate that when students use rich media like mobile social apps in group learning, it can meet their needs well for information exchange with members to promote more knowledge sharing, and high degree of knowledge sharing improves the satisfaction of their learning courses by promoting the collaborative learning effect between members. In other words, media with high media richness are more likely to meet people’s needs and make them satisfied.

Secondly, the results of this study enrich the research of knowledge sharing in higher education. When students use mobile social apps in college courses, not all the functions of these apps can play a role in knowledge sharing. The communication function is the main function that promotes their knowledge sharing in the context of this paper. It can be seen that, when students communicate through mobile social apps, compared with information storing and distribution, real-time and two-way communication can promote knowledge sharing to a greater extent. In addition, this study reveals positive impacts of knowledge sharing on student education. Knowledge sharing can promote students’ satisfaction with a course through collaborative learning indirectly.

Finally, this study finds a mediating role of collaborative learning in the process of students’ course satisfaction through knowledge sharing. This provides support for the idea that, when using mobile social apps, student’ course satisfaction can be driven by the mechanism of collaborative learning. Our results reveal that collaborative learning played a fully mediating role in the relationship between knowledge sharing and course satisfaction. This is consistent with the results that information technology tools have a positive impact on the collaborative learning process (Sangin et al., 2011).

### Practical Implications

This study aims to inspire instructors how to use digital resources to carry out students’ learning. It is hoped to improve teaching by making better use of digital resources to promote collaborative learning and course satisfaction (Ludvigsen et al., 2018). This study has several practical implications for student education. First, according to the empirical results of this study, for those tasks that require teamwork to complete, encouraging communication will help team members to enhance knowledge sharing and promote collaboration, and mobile social tools can provide this communication function. Especially, when it is difficult to face-to-face, such as when digital learning becomes a trend during Post-Pandemic Recovery, the use of mobile social apps can play an important role. Traditionally, many schools and teachers are very opposed to students having too much contact with mobile social apps, thinking that these social media apps are just chat software for privacy, and using too much will distract students. But in the post-epidemic era, guiding students to use mobile social apps correctly can actively promote teaching. Second, teachers can encourage students to use mobile social apps to accomplish learning tasks together in team work. On the one hand, encouraging the sharing of diverse information and knowledge through digital tools can not only improve the efficiency of team work, but also help to meet students’ learning expectations and enable them satisfied with the course. What is more, when designing courses, especially online courses, it is valuable to use digital tools like mobile social apps to build a collaborative learning environment. Secondly, by using its communication and communication functions, we should constantly promote its knowledge sharing and increase shared understanding, so as to achieve students’ satisfaction with the course by improving the level of collaborative learning.

### Limitations and Future Research

This paper has some limitations. First of all, the data of this study are cross-sectional data, which is not objective for the investigation of students’ knowledge sharing and collaborative learning in mobile social apps. In the future, big data technology, such as text mining and machine learning, can be introduced, in order to track students’ dynamic behavior trajectories in mobile social apps, and further excavate the students’ behavior mechanisms. Secondly, the data samples and sources are limited. After the completion of the data collection in this study, 186 valid questionnaire data were obtained through manual identification and deletion. The sample includes students from different provinces and regions in China, as well as a small number of exchange students from other countries. We used this method to minimize common method deviations. But it is true that the number of samples can be larger, not limited to students of the same grade, and groups of different ages can be selected. Finally, in addition to the three functions mentioned in this study, mobile social apps also include information timeliness, analysis, and other features, and future research can explore what other functions will benefit the students’ education. Last but not least, context factors can be considered in exploring the impact of mobile social apps on education, such as culture. This research focuses on how the use of mobile social apps leads to students’ course satisfaction through knowledge sharing and collaborative learning. Future research should also consider how these social apps predict and infer students’ performance, which is another important factor in measuring the effectiveness of course learning. In addition, according to the research of Collazos et al. (2002), the collaborative learning process has multiple dimensions. Future research can further explore whether digital teaching tools will have a differentiated impact on different dimensions of the process, so as to better control the use of it.

## Conclusion

This study discusses the impact of the system characteristics of mobile social apps on students’ learning behavior in the context of group learning and examines how students use mobile social applications to promote collaborative learning and achieve curriculum satisfaction from the perspective of media richness theory and U and G theory. It has been found that the communication function of mobile social apps enables students to share knowledge. This shared understanding can improve students’ course satisfaction by promoting collaborative learning among group members. This study provides useful enlightenment for the introduction and use of mobile social applications in college education. Encouraging the sharing of diverse information and knowledge through digital tools can not only improve the efficiency of team work, but also help to meet students’ learning expectations and enable them satisfied with the course.

## Data Availability Statement

The raw data supporting the conclusions of this article will be made available by the authors, without undue reservation.

## Ethics Statement

Ethical review and approval was not required for the study on human participants in accordance with the local legislation and institutional requirements. Written informed consent for participation was not required for this study in accordance with the national legislation and the institutional requirements. Written informed consent was implied *via* completion of the survey.

## Author Contributions

All authors listed have made a substantial, direct, and intellectual contribution to the work and approved the submitted version.

## Conflict of Interest

RZ is employed by PowerChina Leasing Co., Ltd., Beijing, China.

The remaining authors declare that the research was conducted in the absence of any commercial or financial relationships that could be construed as a potential conflict of interest.

## Publisher’s Note

All claims expressed in this article are solely those of the authors and do not necessarily represent those of their affiliated organizations, or those of the publisher, the editors and the reviewers. Any product that may be evaluated in this article, or claim that may be made by its manufacturer, is not guaranteed or endorsed by the publisher.

## Appendix A

Communication (based on Zhang et al., 2012)

**Table:**

CM1	To what extent do you feel that the communication function of these social mobile apps (like Dingtalk) can help your sharing behaviour be more observed?
CM2	To what extent do you feel that the communication function of these social mobile apps (like Dingtalk) can help knowledge sharing process be more clear?
CM3	To what extent do you feel that the communication function of these social mobile apps (like Dingtalk) can help knowledge structure be more differentiated?

Information Storing (based on Zhang et al., 2012)

**Table:**

IS1	To what extent do you feel that the information storing function of these social mobile apps (like Dingtalk) can help your sharing behaviour be more observed?
IS2	To what extent do you feel that the information storing function of these social mobile apps (like Dingtalk) can help knowledge sharing process be more clear?
IS3	To what extent do you feel that the information storing function of these social mobile apps (like Dingtalk) can help knowledge structure be more differentiated?

Information Distribution (based on Zhang et al., 2012)

**Table:**

ID1	To what extent do you feel that the information distribution function of these social mobile apps (like Dingtalk) can help your sharing behaviour be more observed?
ID2	To what extent do you feel that the information distribution function of these social mobile apps (like Dingtalk) can help knowledge sharing process be more clear?
ID3	To what extent do you feel that the information distribution function of these social mobile apps (like Dingtalk) can help knowledge structure be more differentiated?

Knowledge Sharing (based on Xiao et al., 2017)

**Table:**

KS1	I share success and failure stories about my work in documents with members of my team.
KS2	I share related knowledge obtained from other media (e.g., Websites, newspapers, and journals).
KS3	I share my experience or know-how from work with other team members.
KS4	I provide my knowledge about know-where (where we can find useful things we need) or know-whom (with whom we can discuss) at the request of other team members.
KS5	I share my expertise obtained from my education or training with other team members.

Collaborative learning (based on So and Brush, 2008)

**Table:**

CL1	I felt part of a learning community in my group.
CL2	I actively exchanged my ideas with group members.
CL3	I was able to develop new skills and knowledge from other members in my group.
CL4	I was able to develop problem solving skills through peer collaboration.
CL5	Overall, I am satisfied with my collaborative learning experience in this course.

Satisfaction (based on So and Brush, 2008)

**Table:**

SF1	I was stimulated to do additional readings or research on topics discussed in the course.
SF2	This course was a useful learning experience.
SF3	The diversity of topics in this course prompted me to participate in the discussions.
SF4	My level of learning that took place in this course was of the highest quality.
SF5	Overall, the learning activities and assignments of this course met my learning expectations.
